# Real-World Experience With Mavacamten in SRT-Eligible Obstructive HCM

**DOI:** 10.1016/j.jacadv.2026.102956

**Published:** 2026-07-01

**Authors:** Lusha W. Liang, Hiroo Takayama, Tamim M. Nazif, Linda Gojcaj, Yuichi J. Shimada, Shepard D. Weiner

**Affiliations:** a Division of Cardiology, Department of Medicine, Columbia University Irving Medical Center, New York, New York, USA; b Division of Cardiothoracic Surgery, Department of Surgery, Columbia University Irving Medical Center, New York, New York, USA

**Keywords:** hypertrophic cardiomyopathy, mavacamten, septal reduction therapy

## Abstract

**Background:**

Mavacamten, a cardiac myosin inhibitor, has been shown to improve symptoms and reduce left ventricular outflow tract gradients in obstructive hypertrophic cardiomyopathy (oHCM). However, real-world data regarding longer-term clinical outcomes, safety, and impact on septal reduction therapy (SRT) eligibility are limited.

**Objectives:**

The objective of the study was to evaluate the real-world effectiveness and safety of mavacamten in patients with oHCM who met guideline-based criteria for SRT.

**Methods:**

We retrospectively reviewed patients with oHCM treated with mavacamten at our institution between April 2022 and December 2025 who met guideline-based SRT eligibility criteria. Clinical and echocardiographic outcomes were assessed at baseline and follow-up. Institutional SRT volumes before and after mavacamten approval were compared.

**Results:**

Of the 84 patients treated with mavacamten, 53 met SRT eligibility criteria. The mean age was 67 ± 13 years, and 72% were females. Over a median follow-up of 82 weeks, resting left ventricular outflow tract gradients decreased from 51 ± 39 mm Hg to 4 ± 7 mm Hg and gradients with Valsalva from 79 ± 35 mm Hg to 9 ± 15 mm Hg (both *P* < 0.001). Overall, 49 patients (92%) improved by ≥1 NYHA functional class and no patients remained guideline-eligible for SRT or elected to undergo SRT at the time of most recent follow-up. Transient left ventricular ejection fraction reduction to <50% occurred in 6 patients (11%), and 7 (13%) developed new or recurrent atrial fibrillation. Institutional SRT volume declined significantly after mavacamten approval.

**Conclusions:**

In SRT-eligible oHCM, mavacamten use was associated with sustained hemodynamic and symptomatic improvement and was generally well tolerated in this real-world cohort. Institutional SRT volumes also declined during the post-mavacamten era.

Hypertrophic cardiomyopathy (HCM) is the most common inherited cardiomyopathy, affecting approximately 1:500 to 1:200 individuals worldwide.^1^^,^^2^^,^^3^ The majority of patients with HCM have an obstructive phenotype, characterized by dynamic left ventricular (LV) outflow tract (LVOT) obstruction and symptoms of exertional dyspnea and fatigue.^4^^,^^5^ For many years, treatment of obstructive HCM (oHCM) was focused on nondisease-specific therapies, including beta-blockers, nondihydropyridine calcium-channel blockers, and/or disopyramide.^6^^,^^7^ For those with refractory symptoms despite optimal medical therapy, invasive septal reduction therapy (SRT) with either transaortic septal myectomy (SM) or alcohol septal ablation (ASA) was previously the only definitive therapeutic option.^8^^,^^9^

In recent years, cardiac myosin inhibitors have emerged, which directly target the underlying mechanism of HCM and have shown promise in improving symptoms and LVOT gradients in patients with oHCM.^10^^,^^11^ The VALOR-HCM (A Study to Evaluate Mavacamten in Adults With Symptomatic Obstructive Hypertrophic Cardiomyopathy Who Are Eligible for Septal Reduction Therapy) trial demonstrated that mavacamten use led to a significant reduction in eligibility for SRT or patient decision to proceed with SRT after 16 weeks of treatment.^12^ Results from the long-term extension trial showed sustained improvement with mavacamten.^13^^,^^14^

Despite these promising results, a number of critical questions remain regarding long-term safety and clinical effectiveness of mavacamten. The greatest concern pertains to the impact of mavacamten on LV function. In the long-term extension trial of VALOR-HCM, 11% of patients experienced a reduction in LV ejection fraction (LVEF) to <50%.^13^ Reductions in LVEF to <50% in HCM have been associated with worse long-term outcomes, and it remains unclear whether the reductions in LVEF seen with mavacamten represent favorable remodeling or could be associated with clinical harm in the long-term.^15^ In addition, several studies have raised concern that mavacamten use may be associated with an increased risk of new or recurrent atrial fibrillation (AF).^16^, ^17^, ^18^, ^19^ From an efficacy standpoint, although fewer patients in the mavacamten arm of VALOR-HCM met guideline eligibility for SRT, the same number of patients in each arm ultimately elected to undergo SRT.^12^ Thus, whether mavacamten use could translate into real-world reductions in SRT volume remains uncertain.

In this study, we sought to evaluate the real-world effectiveness and safety of mavacamten in patients with oHCM who were eligible for SRT. We also examined changes in SRT volume and indication at our institution following the introduction of mavacamten.

## Methods

### Patients on mavacamten

This study was conducted with the approval from the Columbia University Irving Medical Center Institutional Review Board. We performed a retrospective review of adult patients with oHCM at our institution who were prescribed mavacamten following its Food and Drug Administration approval between April 2022 and December 2025. Patients were included if they met guideline-based eligibility for SRT. This was defined as patients with NYHA functional class III or IV symptoms despite maximally tolerated medical therapy or those with NYHA functional class II symptoms with exertional syncope or near syncope.^8^ Patients were also required to have a LVOT gradient of ≥50 mm Hg at rest or with Valsalva. SRT was routinely discussed with patients at baseline as part of shared decision-making. Only patients treated with mavacamten for at least 12 weeks were included, given the time needed to achieve steady-state levels of the drug.^10^ Baseline and follow-up clinical characteristics, NYHA functional class, and transthoracic echocardiographic (TTE) parameters were extracted from the electronic medical record. AF surveillance was clinically driven and included symptom-prompted electrocardiograms and, in some cases, ambulatory rhythm monitoring; no standardized monitoring protocol was employed. Follow-up data were collected through December 2025.

### Patients who underwent SRT

To explore temporal changes in SRT referral and procedural patterns at our institution following mavacamten approval, the total volume of patients undergoing SM and ASA from 2017-March 2022 were compared to those who underwent SM and ASA post-mavacamten approval (April 2022 to December 2025). To elucidate surgical trends before and after mavacamten, surgical indications for patients undergoing SM before the approval of mavacamten from 2017-March 2022 were compared to those who underwent myectomy post-mavacamten approval (April 2022 to December 2025).

### Statistics

Statistical analyses were performed using R (R Foundation for Statistical Computing, version 4.5.1). Continuous variables are reported as median (IQR) or mean ± SD, and categorical variables as frequencies and percentages. Normality was assessed using visual inspection and the Shapiro-Wilk test. Paired comparisons of pretreatment and post-treatment parameters were performed using the paired Student’s *t*-test or the Wilcoxon signed rank test, as appropriate. Between-group comparisons were performed using the unpaired t test, Mann-Whitney U test, chi-square test, or Fisher exact test, depending on variable distribution. To account for repeated measures and heterogeneous follow-up duration, longitudinal changes in echocardiographic parameters were additionally evaluated using linear mixed-effects models with time point as a fixed effect and patient-level random intercepts to account for within-patient correlation. Time points included baseline, approximately 12 weeks, and approximately 1 year after mavacamten initiation. Analyses were exploratory and no formal adjustment for multiple comparisons was performed. A *P* value <0.05 was considered statistically significant. Analyses were performed using available data without imputation. Missing data were infrequent and handled using complete-case analysis.

## Results

### Clinical and echocardiographic outcomes following mavacamten initiation

A total of 84 patients on mavacamten were identified, 53 of whom met the guideline criteria for eligibility for SRT (Central Illustration). The mean age of the patients included was 67 ± 13 years, with the majority being females (72%) (Table 1). At baseline, 52 (98%) patients had NYHA functional class III symptoms and the mean LVOT gradient with Valsalva was 79 ± 35 mm Hg. One patient had undergone prior SM and 1 patient had undergone ASA twice. Five (9%) patients were on disopyramide at the time of mavacamten initiation.

**Central Illustration:**
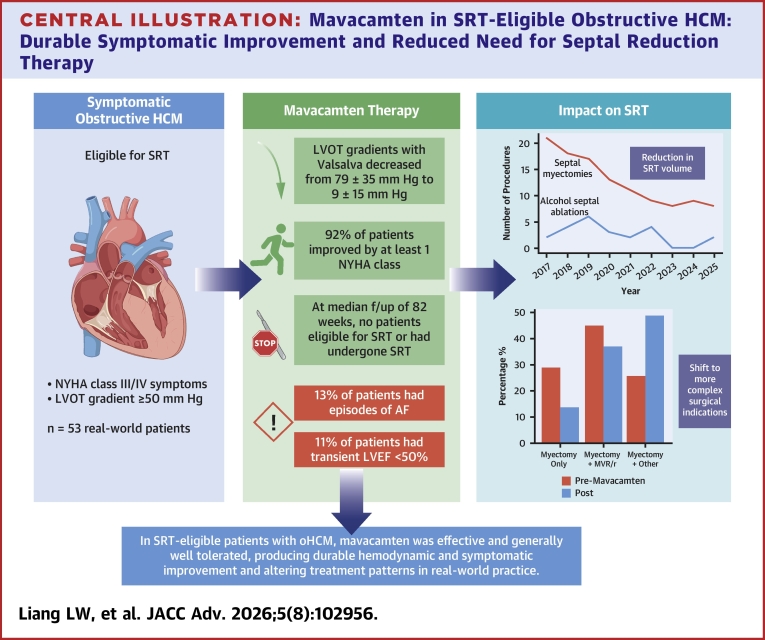
**Mavacamten in SRT-Eligible Obstructive HCM: Durable Symptomatic Improvement and Reduced Need for Septal Reduction Therapy** AF = atrial fibrillation; HCM = hypertrophic cardiomyopathy; LVOT = left ventricular outflow tract; LVEF = left ventricular ejection fraction; MVR/r = mitral valve replacement/repair; oHCM = obstructive hypertrophic cardiomyopathy; SRT = septal reduction therapy.

**Table 1:** Baseline Characteristics (N = 53)

Demographics	
Age (y)	67 ± 13
Male	15 (28)
NYHA functional class	
I	0 (0)
II	1 (2)
III	52 (98)
IV	0 (0)
Medical and family history	
Prior AF	8 (15)
Prior VT/VF	3 (6)
Prior septal myectomy	1 (2)
Prior alcohol septal ablation	1 (2)
Implantable cardioverter defibrillator	4 (8)
Medications	
Beta-blocker	43 (81)
Calcium-channel blocker	19 (36)
Disopyramide	5 (9)
Echocardiographic measurements	
Interventricular septum thickness (mm)	18 ± 4
Left atrial volume index (mL/m^2^)	47 ± 20
Left ventricular outflow tract gradient at rest (mm Hg)	51 ± 39
Left ventricular outflow tract gradient with Valsalva maneuver (mm Hg)	79 ± 35
Left ventricular ejection fraction (%)	67 ± 5
Degree of mitral regurgitation^a^	2 [2-2.5]

Values are n (%). mean ± SD, or median (25th-75th percentile)

AF = atrial fibrillation; VF = ventricular fibrillation; VT = ventricular tachycardia.

a Degree of mitral regurgitation was converted to numerical values according to the following rule: none = 0, trace = 1, trace to mild = 1.5, mild = 2, mild to moderate = 2.5, moderate = 3, moderate to severe = 3.5, severe = 4.

**Figure 1:**
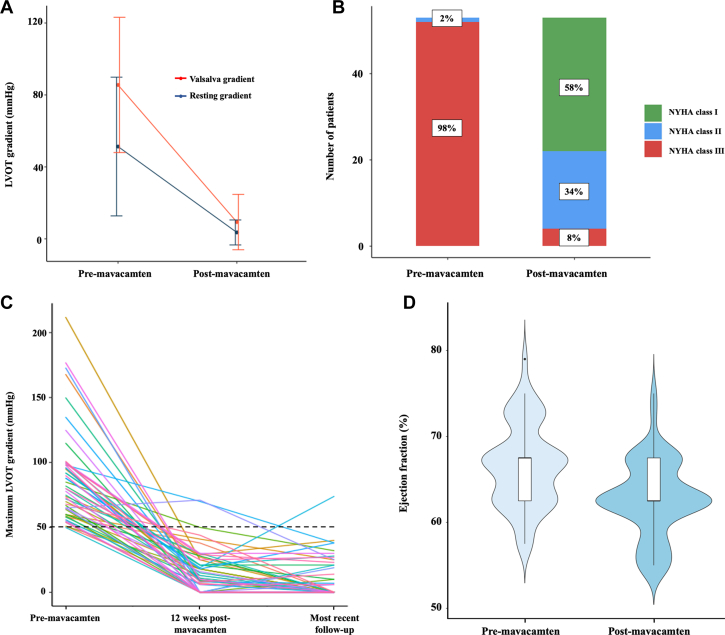
**Clinical and Echocardiographic Outcomes After Mavacamten Initiation** (A) LVOT gradients at rest and with Valsalva before and after mavacamten mean LVOT gradients at rest (blue) and with Valsalva (red) before initiation of mavacamten and at most recent follow-up. Error bars represent SD. (B) NYHA functional class before and after mavacamten distribution of NYHA functional class before mavacamten initiation and at most recent follow-up, demonstrating overall improvement in symptom burden. (C) Individual patient LVOT gradients over time LVOT gradients in individual patients from baseline through most recent follow-up after initiation of mavacamten. (D) LVEF before and after mavacamten mean LVEF before mavacamten initiation and at most recent follow-up. Error bars represent SD. LVOT = left ventricular outflow tract; LVEF = left ventricular ejection fraction.

After being on mavacamten for a median follow-up time of 82 weeks, both the resting gradient and LVOT gradient with Valsalva decreased significantly (Table 2, Figure 1A). Resting LVOT gradients decreased by a mean of 47 mm Hg (95% CI: 37-59 mm Hg; *P* < 0.001) and Valsalva LVOT gradients decreased by a mean of 70 mm Hg (95% CI: 60-80 mm Hg; *P* < 0.001). Longitudinal mixed-effects modeling similarly demonstrated significant reductions in resting and provoked LVOT gradients at both 12-week and 1-year follow-up compared with baseline (all *P* < 0.001) (Supplemental Table 1). The median time from initiation of mavacamten to reduction in maximum gradient of <50 mm Hg was 7 weeks (IQR: 6-9 weeks).

**Table 2:** Clinical and Echocardiographic Characteristics Pre- and Post-Mavacamten

	Pre-Mavacamten (n = 53)	Post-Mavacamten (n = 53)	*P* Value
Medications			
Beta-blocker	43 (81)	38 (72)	0.253
Calcium-channel blocker	19 (36)	13 (25)	0.204
Disopyramide	5 (9)	0 (0)	0.057
NYHA functional class			<0.001
I	0 (0)	31 (58)	-
II	1 (2)	18 (34)	-
III	52 (98)	4 (8)	-
Echocardiographic measurements			
Left atrial volume index (mL/m^2^)	47 ± 20	41 ± 14	0.140
Left ventricular outflow tract gradient at rest (mm Hg)	51 ± 39	4 ± 7	<0.001
Left ventricular outflow tract gradient with Valsalva (mm Hg)	79 ± 35	9 ± 15	<0.001
Left ventricular ejection fraction (%)	67 ± 5	63 ± 5	<0.001
Degree of mitral regurgitation^a^	2 [2-2.5]	2 [1-2]	0.148
E/e’ average ratio	17 ± 8	14 ± 6	0.043
RVSP	30 ± 12	29 ± 10	0.613

Values are n (%), mean ± SD, or median (25th-75th percentile).

a Degree of mitral regurgitation was converted to numerical values according to the following rule: none = 0, trace = 1, trace to mild = 1.5, mild = 2, mild to moderate = 2.5, moderate = 3, moderate to severe = 3.5, severe = 4; RVSP = right ventricular systolic pressure.

At the time of most recent follow-up, 31 (58%) patients had NYHA functional class I symptoms and 18 (34%) patients had NYHA functional class II symptoms (Table 2, Figure 1B). Four (8%) patients continued to have NYHA functional class III symptoms but had LVOT gradients at rest and with Valsalva <50 mm Hg. One patient’s most recent TTE showed a rise in the LVOT gradient with Valsalva to ≥50 mm Hg (Figure 1C). However, this patient had been septic from a urinary infection at the time of the TTE, so no changes were made to the mavacamten dosing.

Indices of diastolic function showed modest improvement, with a reduction in E/e′ (17 ± 8 vs 14 ± 6; *P* = 0.043). Disopyramide was discontinued during follow-up in all patients. In addition, after being started on mavacamten, beta-blocker and/or calcium-channel blocker therapy were tapered or discontinued in 27 (51%) patients (Table 2).

SRT was discussed with patients at baseline and reconsidered during follow-up in those with persistent symptoms or residual LVOT obstruction; however, symptom and hemodynamic improvement during follow-up was accompanied by resolution of guideline-based SRT eligibility in all patients at the most recent evaluation.

### Safety of mavacamten

There was a mean decrease of 4% (95% CI: 2% to 5%; *P* < 0.001) in LVEF after mavacamten use at the most recent follow-up (Table 2, Figure 1D, Supplemental Table 1). Six patients (11%; 95% CI: 4.7% to 23.7%) experienced a reduction in LVEF to <50%. In all cases, therapy was temporarily discontinued and resumed at a lower dose following recovery of LVEF (Table 3). Two of these 6 patients (33%) experienced a decline in LVEF in the setting of AF with rapid ventricular response (RVR) (patients 2 and 4). Patient 2 had an episode of AF with RVR 6 months after treatment initiation, with reduction in LVEF to 35% and associated symptoms of acute decompensated heart failure. LVEF normalized following temporary discontinuation of mavacamten and direct current cardioversions (DCCVs), and therapy was resumed at a reduced dose. Although AF recurred without further LVEF decline, the patient ultimately underwent two AF ablations for persistent symptomatic AF. Patient 4 developed heart failure symptoms after 4 months of mavacamten and was found to be in AF with RVR with LVEF 40% and apical hypokinesis on TTE, suggesting a diagnosis of takotsubo/stress cardiomyopathy. After DCCV and discontinuation of mavacamten, the patient’s LVEF recovered and mavacamten was resumed. However, the patient had recurrence of persistent symptomatic AF and underwent AF ablation, followed by DCCV, and atrioventricular nodal ablation. Subsequent course was complicated by stroke, pneumonia, and recurrent hospitalizations for hypoxic respiratory failure and the patient died 12 months after treatment initiation. The remaining four patients were asymptomatic at the time of LVEF decline with reduced systolic function identified on routine surveillance TTE.

**Table 3:** Patients With Reduction in LVEF to Less Than 50%

Patient	Baseline LVEF	Time to LVEF <50%	LVEF Nadir	Symptoms During LVEF <50%	Dose Adjustment	Most Recent LVEF	Most Recent LVOT Gradient	NYHA Functional Class^a^
Patient 1	60%-65%	2 mo	45%-50%	Asymptomatic	5 → 2.5 mg	60%-65%	7 mm Hg	III → II
Patient 2	60%-65%	12 mo	35%	AF, heart failure	5 → 2.5 mg	55%	10 mm Hg	III → II
Patient 3	60%-65%	5 mo	45%-50%	Asymptomatic	5 → 2.5 mg	60%-65%	9 mm Hg	III → I
Patient 4	55%-60%	4 mo	40%	AF, heart failure	5 → 2.5→ 5 mg	55%-60%	10 mm Hg	III→deceased
Patient 5	55%-60%	5 mo	40%-45%	Asymptomatic	5 → 2.5 mg	55%	18 mm Hg	III → I
Patient 6	60%-65%	9 mo	40%-45%	Asymptomatic	5 → 2.5 mg	55%-60%	19 mm Hg	III → II

LVEF = left ventricular ejection fraction; LVOT = left ventricular outflow tract; other abbreviation as in Table 1.

a Change in NYHA functional class before mavacamten to most recent.

Regarding AF overall, 8 (15%) patients had a pre-existing diagnosis of AF at the time of mavacamten initiation (Table 4). Among these, 5 (63%; 95% CI: 25.9% to 89.8%) experienced AF recurrence. One episode self-terminated (Patient 1), 2 were successfully treated with DCCV (patients 3 and 5), and 2 were associated with LVEF reduction as described previously (patients 2 and 4). Patient 6 had longstanding asymptomatic permanent AF, and no further treatment was pursued. Patient 7, who had undergone two AF ablations before starting mavacamten, had no recurrence of AF. In addition, 2 patients (4%; 95% CI: 0.8% to 16.4%) developed new-onset AF after starting mavacamten (patients 9 and 10). One developed symptomatic AF at 6 months and underwent successful DCCV (patient 9), whereas the other developed asymptomatic AF at 2 months and was managed with beta blocker therapy alone (Patient 10).

**Table 4:** Patients With Recurrent or New-Onset AF After Starting Mavacamten

Patient #	Age (y), Sex	AF Treatment Pre-Mavacamten	Time to AF	AF Treatment Post-Mavacamten	∗NYHA Functional Class
1	77, F	Paroxysmal AF→AF ablation, disopyramide	8 mo	Self-terminated	III→II
2	74, F	Persistent AF→DCCV x2, disopyramide	6 mo	DCCV x3, AF ablation x2, amiodarone	III→II
3	78, F	Paroxysmal AF→DCCV x 1	2 mo	DCCV x 2	III→I
4	72, F	Paroxysmal AF→DCCV x 2	2 mo	DCCV x 1, AF ablation, AVN ablation	III → deceased
5	21, F	Paroxysmal AF on sotalol	1 mo	DCCV x 2	III→II
6	72, F	Permanent asymptomatic AF	-	Asymptomatic, no treatment	III→I
7	64, M	Paroxysmal AF→ AF ablation x 2	-	No recurrence of AF	III→I
8	86, F	Paroxysmal AF	-	No recurrence of AF	III→II
9	88, M	No prior AF	6 mo	DCCV x 1	III→II
10	73, M	No prior AF	2 mo	Asymptomatic, no treatment	III→I

AVN = atrioventricular node; DCCV = direct current cardioversion; other abbreviation as in Table 1.

∗ Change in NYHA functional class before mavacamten to most recent.

### Surgical myectomies and alcohol septal ablations

**Figure 2:**
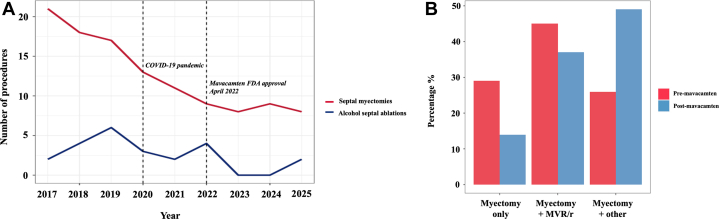
**Institutional Procedural Trends Before and After Mavacamten Approval** (A) Annual septal reduction therapy volume, 2017-2025. Number of septal myectomies and alcohol septal ablations performed annually from 2017 through 2025, illustrating procedural trends before and after mavacamten availability. (B) Types of septal myectomy performed before and after mavacamten distribution of septal myectomy subtypes performed before and after mavacamten availability, demonstrating shifts in surgical case complexity. FDA = Food and Drug Administration; MVR/r = mitral valve replacement/repair.

Between 2017 and 2020, an average of 17.3 SMs and 3.8 ASAs were performed annually at our institution (Figure 2A). Surgical and procedural volumes declined in 2020 to 2021, coinciding with the COVID-19 pandemic. Following the approval of mavacamten, annual volumes decreased further, with an average of 8.5 SMs and 1.5 ASAs performed per year between April 2022 and 2025. Despite the pandemic-related decline, SM volume significantly decreased after mavacamten approval (median 8.5 [IQR: 1] post-mavacamten vs 17 [IQR: 5] pre-mavacamten; *P* = 0.037). ASA volume also declined (median 1 [IQR: 2.5] vs 3 [IQR: 2]; *P* = 0.047). In addition to the decline in overall case volume for septal myectomies, surgical indications shifted: the proportion of isolated septal myectomies decreased, whereas the proportion performed in conjunction with valvular procedures (including mitral valve repair/replacement) and/or coronary artery bypass grafting increased (*P* = 0.002) (Figure 2B). Among the cases involving concomitant procedures other than mitral valve repair/replacement (“myectomy + other” in Figure 2B), the primary indication for surgery was SM in 9/21 cases (43%) pre-mavacamten and 6/17 cases (35%) post-mavacamten, with the remaining cases primarily driven by other cardiac abnormalities, including multivessel coronary artery disease, severe aortic stenosis, aortic aneurysm, and endocarditis.

## Discussion

In this real-world cohort of patients with symptomatic oHCM, mavacamten use was associated with substantial reductions in LVOT gradients and improvements in symptoms in the majority of patients, with durable effects over a median follow-up of 82 weeks. Notably, no patients remained guideline-eligible for SRT at the time of the most recent follow-up, and none had elected to proceed with SRT. These findings extend the results of VALOR-HCM into routine clinical practice and suggest that cardiac myosin inhibition is associated with meaningful changes in therapeutic trajectories in selected patients with oHCM. Importantly, this study is among the first to demonstrate a temporal decline in SRT volumes following the introduction of mavacamten, accompanied by a shift toward more complex surgical referrals.

Although efficacy was consistent, safety considerations remain. Six patients (11%) experienced a temporary decline in LVEF to <50%, and there was a decline in the mean LVEF overall. Under the Risk Evaluation and Mitigation Strategy program for mavacamten, routine surveillance for systolic dysfunction is mandated; pooled Risk Evaluation and Mitigation Strategy data with up to 22 months of follow-up have reported a 4.6% incidence of LVEF <50%, although this estimate may be influenced by survival bias.^20^ In contrast, a recent multicenter study reported LVEF <50% in 8% of patients over a median of 7.8 months of therapy, more closely aligning with the incidence observed in our cohort.^19^

In HCM, reductions in LVEF, particularly below 50%, have traditionally been associated with advanced fibrosis, adverse remodeling, and progression to end-stage disease.^15^ However, current evidence suggests that mavacamten may have more favorable effects on cardiac remodeling. Several studies have demonstrated improvements in echocardiographic diastolic parameters with mavacamten, including left atrial volume index and E/e’, which have been previously shown to predict long-term outcomes in HCM.^21^, ^22^, ^23^ In addition, the cardiac magnetic resonance imaging substudy of EXPLORER-HCM, which included 17 patients on mavacamten and 18 patients on placebo, found that mavacamten use led to substantial LV remodeling with greater reduction in mean LV mass index compared to placebo.^24^ Although the evidence has been favorable thus far, there remains a need for longer-term imaging studies and clinical follow-up in larger patient cohorts.

AF was observed in 7 patients (13%; 95% CI: 5.9% to 26.0%) in our cohort, consistent with prior reports in patients receiving mavacamten.^16^, ^17^, ^18^, ^19^ However, most events reflected recurrence in patients with pre-existing AF, with new-onset AF occurring at rates comparable to those reported in HCM populations not treated with mavacamten (approximately 2% to 4% per year).^25^ Despite the occurrence of AF, many patients continued to demonstrate lower LVOT gradients and improved symptoms during follow-up. Given that patients with LVOT obstruction rely more on left atrial contraction for LV filling, reduction or elimination of LVOT gradients with mavacamten may allow patients with HCM to be less symptomatic, even when they are in AF.^25^ In contrast, the 2 patients who developed a reduction in LVEF in relation to AF with RVR presented to the hospital in heart failure, and both required multiple interventions for rhythm control for symptomatic AF. A multicenter study reported that of the patients who experienced a reduction in LVEF <50% while receiving mavacamten, more than two-thirds had concomitant AF, raising the possibility of increased susceptibility to systolic dysfunction in this subgroup.^19^ The clinical and echocardiographic characteristics of patients who may be more at risk for AF and LVEF reduction with mavacamten remain incompletely understood. Collectively these findings highlight our evolving understanding of the effects of mavacamten on AF and LV function and underscore a need for further mechanistic studies and vigilant long-term monitoring for arrhythmias and systolic dysfunction.

Beyond individual patient outcomes, our findings suggest temporal changes in referral patterns and procedural practice during the post-mavacamten era. Both SM and ASA volumes decreased; moreover, the patients who did proceed to surgery were more likely to have concomitant structural abnormalities, such as mitral or aortic valve disease, requiring operative correction. These observations may reflect an evolving and potentially more specialized role of surgery in the era of myosin inhibitors, and emphasizes the importance of multidisciplinary decision-making and a “Heart Team approach” to align treatment strategies with individual patient characteristics and preferences. Indeed, in our cohort there was 1 patient who had a residual LVOT gradient of ≥50 mm Hg at the time of most recent follow-up, and other studies have found that about 15% to 35% of patients do not respond or have an incomplete response to mavacamten.^10^^,^^20^^,^^26^ Conversely, SM has been shown to relieve symptoms in >90% of patients by ≥1 NYHA functional class, and has been associated with improved long-term survival in observational studies.^27^^,^^28^ Observational studies have also found that SM was associated with decreased risk for new-onset AF.^29^ Cost-effectiveness also warrants attention, as most patients will require lifelong therapy, raising substantial economic considerations compared to one-time definitive interventions such as surgery, particularly for younger patients.^30^ Finally, as occurred in Europe with the rise of ASA, if SM volumes continue to decline, then surgical expertise and training will certainly be impacted. This has important implications for patient care, as higher institutional volume has been associated with superior patient outcomes.^31^

Future work should focus on identifying clinical, imaging, and biomarker predictors of response to mavacamten to better personalize therapy and guide dose titration and frequency of monitoring. As cardiac myosin inhibitors become increasingly integrated into HCM care, precision phenotyping may be essential to determine which patients derive durable benefit vs those who may require earlier referral for SRT.

### Study Limitations

Our study has several limitations. Given the observational single-arm design, causality cannot be inferred and the potential contributions of regression to the mean, referral bias, evolving treatment practices, and secular trends cannot be excluded. Because this study included only patients treated with mavacamten, those referred directly for SRT were not captured, introducing potential selection bias. The relatively older age of our cohort may also influence treatment preferences, as older patients may be less likely to pursue invasive therapies such as SM. AF surveillance was not standardized and was primarily clinically driven, which may have led to underdetection of asymptomatic or subclinical events. Outside of the Valsalva maneuver, other provocation maneuvers (such as postprandial or postexercise echocardiography) were not uniformly performed, and given the dynamic nature of LVOT obstruction, gradients may be underestimated. Finally, procedural trends should be interpreted cautiously given potential confounding from COVID-era disruptions, referral variation, and evolving surgical selection patterns.

## Conclusions

In summary, mavacamten was associated with a reduction in the need for SRT in a real-world cohort of patients with oHCM, with durable improvements in LVOT gradients and symptoms in the majority of patients. At a systems level, these patient-level observations were accompanied by a decline in institutional SRT volume and a shift toward more complex surgical indications, suggesting potential changes in contemporary treatment patterns. These findings suggest that the benefits observed in clinical trials may also be observed in routine practice settings, supporting mavacamten as an important therapeutic option for many patients with oHCM. However, the occurrence of AF and reductions in LVEF in a subset of patients highlights the need for careful patient selection and ongoing monitoring for long-term safety. Ongoing evaluation will be essential to clarify its long-term effects on ventricular remodeling, arrhythmia burden, and systolic function, as well as to determine its cost-effectiveness and role within comprehensive care pathways.

Perspectives **COMPETENCY IN MEDICAL KNOWLEDGE:** Mavacamten use was associated with substantial and durable reductions in LVOT gradients and symptom burden in patients with oHCM who meet the guideline-based criteria for SRT. In this real-world cohort, no patients remained guideline-eligible for SRT at a median follow-up time of 82 weeks. Importantly, this study is among the first to demonstrate a quantifiable decline in SM and ASA volumes following the introduction of mavacamten, accompanied by a shift toward more complex surgical referrals. These findings suggest that, in appropriately selected patients, cardiac myosin inhibition may influence treatment pathways and potentially reduce reliance on invasive intervention, while requiring careful monitoring for AF and reversible reductions in LVEF.

**TRANSLATIONAL OUTLOOK:** Our findings highlight the need for prospective, multicenter studies with standardized imaging and rhythm monitoring to better define predictors of response to mavacamten and to clarify its effects on AF risk and ventricular function. In addition, long-term comparative effectiveness studies are needed to determine the relative roles of myosin inhibitors and septal reduction therapies, including durability of benefit, impact on clinical outcomes, and cost-effectiveness. More broadly, the observed decline in SRT use underscores the need to understand how evolving use of myosin inhibitors may influence referral patterns, procedural expertise, and training in hypertrophic cardiomyopathy care.

## Funding support and author disclosures

This work was supported by the 10.13039/100000002National Institutes of Health (R01 HL157216 and R01 HL168382 to Yuichi J. Shimada), the 10.13039/100000968American Heart Association (2 National Clinical and Population Research Awards, 1 Career Development Award, and 1 Transformational Project Award to Dr Shimada), Korea Institute of Oriental Medicine (W25001 to Dr Shimada), Feldstein Medical Foundation (to Dr Shimada), Columbia University Irving Medical Center Precision Medicine Pilot Award (to Dr Shimada), and Columbia University Irving Medical Center Marjorie and Lewis Katz Cardiovascular Research Prize (to Dr Shimada). Dr Shimada is a consultant for Bristol Myers Squibb and Moderna, Japan. Dr Weiner is a consultant for Bristol Myers Squibb and Cytokinetics. All other authors have reported that they have no relationships relevant to the contents of this paper to disclose.

## References

[1] Maron B.J., Gardin J.M., Flack J.M., Gidding S.S., Kurosaki T.T., Bild D.E. (1995). Prevalence of hypertrophic cardiomyopathy in a general population of young adults. Circulation.

[2] Maron B.J., Desai M.Y., Nishimura R.A. (2022). Management of hypertrophic Cardiomyopathy. J Am Coll Cardiol.

[3] Semsarian C., Ingles J., Maron M.S., Maron B.J. (2015). New perspectives on the prevalence of hypertrophic cardiomyopathy. J Am Coll Cardiol.

[4] Veselka J., Anavekar N.S., Charron P. (2017). Hypertrophic obstructive cardiomyopathy. Lancet.

[5] Maron B.J. (2018). Clinical course and management of hypertrophic cardiomyopathy. N Engl J Med.

[6] Ommen S.R., Mital S., Burke M.A. (2020). 2020 AHA/ACC Guideline for the diagnosis and treatment of patients with hypertrophic cardiomyopathy. Circulation.

[7] Liang L.W., Lumish H.S., Sewanan L.R. (2024). Evolving strategies for the management of obstructive hypertrophic cardiomyopathy. J Card Fail.

[8] Ommen S.R., Ho C.Y., Asif I.M. (2024). 2024 AHA/ACC/AMSSM/HRS/PACES/SCMR Guideline for the management of hypertrophic cardiomyopathy: a report of the American Heart Association/American College of Cardiology Joint Committee on Clinical Practice Guidelines. J Am Coll Cardiol.

[9] Nguyen A., Schaff H.V., Hang D. (2019). Surgical myectomy versus alcohol septal ablation for obstructive hypertrophic cardiomyopathy: a propensity score–matched cohort. J Thorac Cardiovasc Surg.

[10] Olivotto I., Oreziak A., Barriales-Villa R. (2020). Mavacamten for treatment of symptomatic obstructive hypertrophic cardiomyopathy (EXPLORER-HCM): a randomised, double-blind, placebo-controlled, phase 3 trial. Lancet.

[11] Maron M.S., Masri A., Nassif M.E. (2024). Aficamten for symptomatic obstructive hypertrophic cardiomyopathy. N Engl J Med.

[12] Desai M.Y., Owens A., Geske J.B. (2022). Myosin inhibition in patients with obstructive hypertrophic cardiomyopathy referred for septal reduction therapy. J Am Coll Cardiol.

[13] Desai M.Y., Owens A., Wolski K. (2023). Mavacamten in patients with hypertrophic Cardiomyopathy referred for septal reduction: week 56 results from the VALOR-HCM randomized clinical trial. JAMA Cardiol.

[14] Desai M.Y., Wolski K., Owens A. (2025). Mavacamten in patients with hypertrophic Cardiomyopathy referred for septal reduction: week 128 results from VALOR-HCM. Circulation.

[15] Maron B.J., Rowin E.J., Udelson J.E., Maron M.S. (2018). Clinical spectrum and management of heart failure in hypertrophic cardiomyopathy. JACC Hear Fail.

[16] Liang L.W., Lumish H.S., Shimada Y.J., Weiner S.D. (2024). Incidence and recurrence of atrial fibrillation among patients with obstructive hypertrophic cardiomyopathy treated with mavacamten: a single-center experience. Clin Res Cardiol.

[17] Castrichini M., Alsidawi S., Geske J.B. (2024). Incidence of newly recognized atrial fibrillation in patients with obstructive hypertrophic cardiomyopathy treated with Mavacamten. Hear Rhythm.

[18] Boyle T.A., Reza N., Hyman M. (2025). Atrial fibrillation in patients receiving mavacamten for obstructive hypertrophic cardiomyopathy: real-world incidence, management, and outcomes. JACC Clin Electrophysiol.

[19] Nguyen O., Wiedrick J., Massera D. (2025). Incidence and outcomes of atrial fibrillation and systolic dysfunction in patients receiving mavacamten for obstructive hypertrophic cardiomyopathy: a multicenter study. J Card Fail.

[20] Desai M.Y., Seto D., Cheung M. (2025). Mavacamten: real-world experience from 22 months of the risk Evaluation and Mitigation Strategy Program. Circ Hear Fail.

[21] Hegde S.M., Lester S.J., Solomon S.D. (2021). Effect of mavacamten on echocardiographic features in symptomatic patients with obstructive hypertrophic cardiomyopathy. J Am Coll Cardiol.

[22] Cremer P.C., Geske J.B., Owens A. (2022). Myosin inhibition and left ventricular diastolic function in patients with obstructive hypertrophic cardiomyopathy referred for septal reduction therapy: insights from the VALOR-HCM Study. Circ Cardiovasc Imaging.

[23] Wessly P., Lazzara G.E., Buergler J.M., Nagueh S.F. (2023). Early observations on effects of mavacamten on left atrial function in obstructive hypertrophic cardiomyopathy patients. JACC Cardiovasc Imaging.

[24] Saberi S., Cardim N., Yamani M. (2021). Mavacamten favorably impacts cardiac structure in obstructive hypertrophic cardiomyopathy. Circulation.

[25] Vaidya K., Semsarian C., Chan K.H. (2017). Atrial fibrillation in hypertrophic cardiomyopathy. Hear Lung Circ.

[26] Braunwald E., Saberi S., Abraham T.P., Elliott P.M., Olivotto I. (2023). Mavacamten: a first-in-class myosin inhibitor for obstructive hypertrophic cardiomyopathy. Eur Heart J.

[27] Maron B.J., Dearani J.A., Ommen S.R. (2015). Low operative mortality achieved with surgical septal myectomy: role of dedicated hypertrophic cardiomyopathy centers in the management of dynamic subaortic obstruction. JACC.

[28] Ommen S.R., Maron B.J., Olivotto I. (2005). Long-term effects of surgical septal myectomy on survival in patients with obstructive hypertrophic cardiomyopathy. J Am Coll Cardiol.

[29] Rowin E.J., Cooper C., Carrick R.T., Tsoi M., Marron B.J., Maron M.S. (2022). Ventricular septal myectomy decreases long-term risk for atrial fibrillation in patients with hypertrophic cardiomyopathy. Am J Cardiol.

[30] Beinfeld M., Nhan E., Rind D.M. (2022). Mavacamten for hypertrophic cardiomyopathy: effectiveness and value. J Manag Care Spec Pharm.

[31] Holst K.A., Schaff H.V., Smedira N.G. (2022). Impact of hospital volume on outcomes of septal myectomy for hypertrophic cardiomyopathy. Ann Thorac Surg.

