# Supplementary material for: Real-World Experience With Mavacamten in SRT-Eligible Obstructive HCM
Source: JACC Adv. 2026 Jul 1;5(8):102956. doi: 10.1016/j.jacadv.2026.102956 (PMC13355183; doi:10.1016/j.jacadv.2026.102956)
Supplement: Supplemental_Material [file mmc1.docx]

| **Parameter** | **12-week change vs. Baseline** | **p-value** | **1-year change vs. Baseline** | **p-value** |
| --- | --- | --- | --- | --- |
| **Resting LVOT gradient** | −41 mmHg (95% CI: −52 to −31) | <0.0001 | −47 mmHg (95% CI: −57 to −37) | <0.0001 |
| **Valsalva LVOT gradient** | −65 mmHg (95% CI: −74 to −56) | <0.0001 | −70 mmHg (95% CI: −79 to −61) | <0.0001 |
| **LVEF** | −3% (95% CI: −5 to −2) | <0.0001 | −4% (95% CI: −5 to −2) | <0.0001 |

**Supplemental Table 1: Longitudinal Mixed-Effects Modeling of Echocardiographic Parameters Following Mavacamten Initiation**

Abbreviations: LVEF: left ventricular ejection fraction; LVOT: left ventricular outflow tract
